# A Complex Story: Universal Preference vs. Individual Differences Shaping Aesthetic Response to Fractals Patterns

**DOI:** 10.3389/fnhum.2016.00213

**Published:** 2016-05-24

**Authors:** Nichola Street, Alexandra M. Forsythe, Ronan Reilly, Richard Taylor, Mai S. Helmy

**Affiliations:** ^1^School of Psychology, Sport and Exercise Faculty of Health Sciences, Staffordshire UniversityStoke-on-Trent, UK; ^2^School of Psychology, University of LiverpoolLiverpool, UK; ^3^Department of Computer Science, National University of IrelandMaynooth, Ireland; ^4^Department of Physics, University of OregonEugene, OR, USA; ^5^Department of Psychology, Menoufia UniversityMenoufia, Egypt

**Keywords:** aesthetics, complexity, fractals, preference, culture, gender

## Abstract

Fractal patterns offer one way to represent the rough complexity of the natural world. Whilst they dominate many of our visual experiences in nature, little large-scale perceptual research has been done to explore how we respond aesthetically to these patterns. Previous research (Taylor et al., 2011) suggests that the fractal patterns with mid-range fractal dimensions (FDs) have universal aesthetic appeal. Perceptual and aesthetic responses to visual complexity have been more varied with findings suggesting both linear (Forsythe et al., 2011) and curvilinear (Berlyne, 1970) relationships. Individual differences have been found to account for many of the differences we see in aesthetic responses but some, such as culture, have received little attention within the fractal and complexity research fields. This two-study article aims to test preference responses to FD and visual complexity, using a large cohort (*N* = 443) of participants from around the world to allow universality claims to be tested. It explores the extent to which age, culture and gender can predict our preferences for fractally complex patterns. Following exploratory analysis that found strong correlations between FD and visual complexity, a series of linear mixed-effect models were implemented to explore if each of the individual variables could predict preference. The first tested a linear complexity model (likelihood of selecting the more complex image from the pair of images) and the second a mid-range FD model (likelihood of selecting an image within mid-range). Results show that individual differences can reliably predict preferences for complexity across culture, gender and age. However, in fitting with current findings the mid-range models show greater consistency in preference not mediated by gender, age or culture. This article supports the established theory that the mid-range fractal patterns appear to be a universal construct underlying preference but also highlights the fragility of universal claims by demonstrating individual differences in preference for the interrelated concept of visual complexity. This highlights a current stalemate in the field of empirical aesthetics.

## Introduction

The reduction of aesthetic responses to perceptual stimuli to basic formuli has been a goal of perceptual research since its inception. Birkhoff (1933) argued that order was a positive contributor to aesthetic evaluation, that preferences are driven by the need to experience stability in an image and, as a general rule, visual complexity contributed negatively to the aesthetic experience. Gombrich (1984) extended this opinion by stating that aesthetic appeal lay at the mid-point between complete monotony and total unintelligible chaos.

## Beauty and Complexity

**Figure 1:**
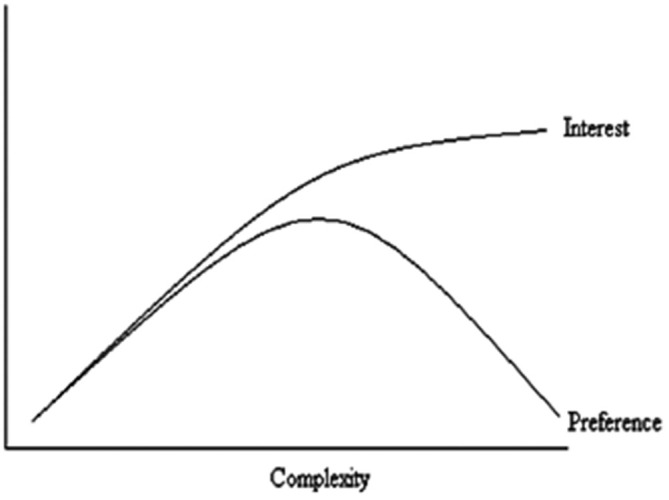
**Berlyne’s hedonic experience and complexity**.

Exploring the impact of visual complexity in aesthetic experience has stumbled somewhat on the lack of a unified definition of visual complexity. Complexity arises in situations where an increasing number of independent variables begin interacting in unpredictable interdependent ways (Forsythe et al., 2011). One issue the field of visual complexity faces is the inconsistency of stimulus used to explore the relationship between complexity and visual experience. As outlined in the above discussions, some studies use computer or hand generated stimulus, which increases in complexity by the number of objects, amounts of turns, or presence of symmetry. Others use photographs or nature, art or websites to explore responses to complexity in a more ecologically applicable way. There are a wide variety of approaches with which to gather complexity ratings or rankings from stimulus. Whilst some employ human judgments others have attempted to develop advanced quantitative measures to provide this information, as with computational compression techniques. Berlyne’s (1970) curvilinear hypothesis has received the most attention in this regard. Berlyne (1970) argued that preference increases linearly with complexity until an optimum level of visual arousal is reached. When visual stimuli are of low complexity (i.e., simple), or highly complex, aesthetic preference will also be low (Figure 1).

Berlyne’s theory has received mixed support because it does not predict the point of the preference cusp (Krupinski and Locher, 1988; Martindale et al., 1990). Furthermore, results consistent with the Berlyne hypothesis were often limited by sample size and biased by familiarity with an image (Forsythe et al., 2008). To further explore this relationship with an unbiased sample, Forsythe et al. (2011) applied compression measures of complexity when standardizing 800 artistic images for visual complexity and beauty. Automated measures have also extended our understanding of the relationship between complexity and beauty. In particular, compression measures of visual complexity offer one method to capture the complexity of an image and a number of different compression measures exist that are suitable to different types of image. A compressed image consists of a string of numbers that represent the organization of that picture. This string is a measure of information content (Donderi, 2006). When the image contains few elements or is more homogenous in design, there are few message alternatives and as such the file string contains mostly numbers to be repeated. A more complex picture will have more image elements and these elements will be less predictable: the file string will be longer and contain an increasing number of alternatives. Using this method, Forsythe et al. (2011) found that when familiarity was controlled for, the relationship between beauty and visual complexity was more linear in character, with preference taking a different pattern to the curvilinear pattern originally proposed by Berlyne (1970). Despite the wealth of current findings, there is still little consensus about what complexity is and how it should be defined and measured (Forsythe, 2009). The lack of consistency in what it is to be complex in a stimulus means that the field cannot move forward in a unified way. Some argue that complexity as one concept does not exist, and instead we need to explore it as a multidimensional construct, that many different sub-sectors of complexity exist instead of one overarching definition (Forsythe et al., 2011).

**Figure 2:**
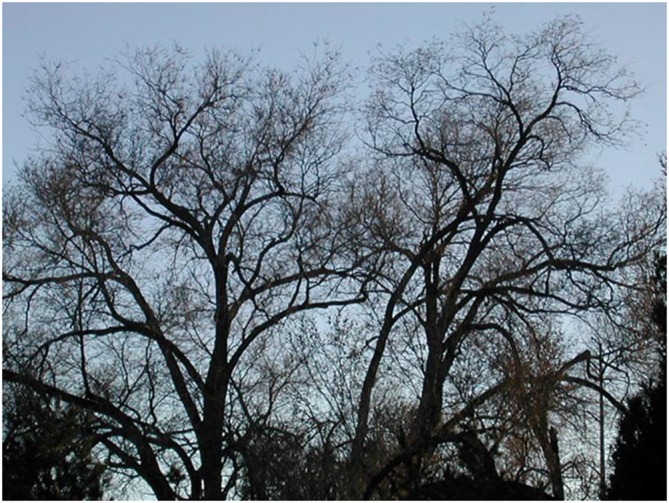
**Fractal landscape**.

**Figure 3:**
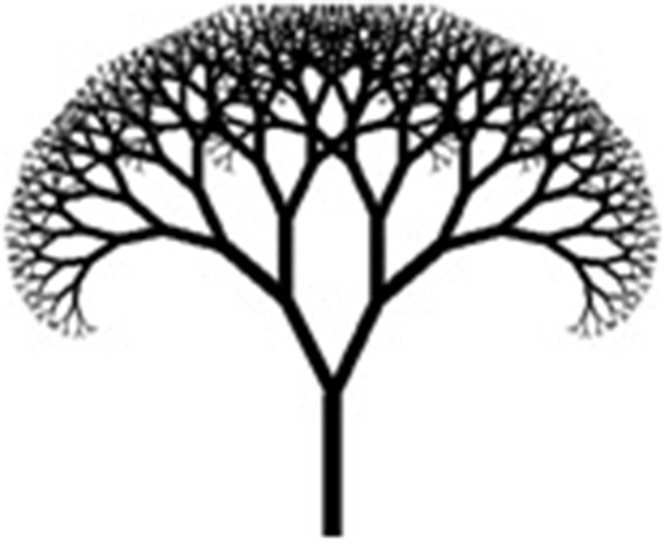
**Mathematical fractal**.

Fractal dimension (FD) measures the relative amounts of coarse and fine structure in a fractal pattern and has been proposed as a new method of quantifying the complexity of nature and the relationship between the universality and individual differences (Forsythe et al., 2011). The fractal, a self-similar pattern that reoccurs on finer and finer scales, has been demonstrated to characterize many of the visual patterns in the natural world (Figures 2, 3). Fractal analysis has been successfully used in quantifying the complex structure exhibited by many natural patterns and has captured the imagination of scientists and artists alike, being characterized as “fingerprints of nature” and “the new aesthetics”. It is thought that fractals tap into specialist cognitive modules that have developed to moderate information about living things (Wilson, 1984) and that such modules are linked with emotional regulation and the reduction of physiological stress (Taylor et al., 2006, 2011). Hagerhall et al. (2008, 2015) report that viewing fractal patterns elicited high alpha in areas of the brain concerned with attention and visuo-spatial processing providing support for the idea that training using fractal shapes could help the development of perceptual concepts of the natural, stimulate biophilic responses and trigger aesthetic interest and restorative responses (Joye, 2005, 2006). In line with this, The Savannah hypothesis suggests that spontaneous emotional responses are demonstrated towards landscapes that are positive for survival or instincts which may account for the heightened preference for scenes falling within the mid-range of the fractal spectrum (Appleton, 1975; Orians, 1980; Falk and Balling, 2009).

Fractal geometry has established its usefulness in understanding the degeneration of artworks by artists subsequently diagnosed with neurological deterioration (Forsythe et al., 2005) and in determining the authenticity of major works of art. Taylor et al. (1999) examined the work of Jackson Pollock and determined that Pollock was generating paintings with a high FD and that Pollock was able to fine-tune the dimension of his paintings. Taylor’s work may also be useful in addressing some of the shortcomings of the Berlyne (1970) hypothesis.

## Cross Cultural Differences

Spehar et al. (2003) report the presence of three categories with respect to aesthetic preference for FD (Spehar et al., 2003; Spehar and Taylor, 2013). Humans are consistent in their low preference for patterns with dimensions between (1.1–1.2) and (1.6–1.9), preferring fractal images in the 1.3–1.5 FD range. This effect is constant regardless of whether these fractals were generated by mathematics (Figure 3), artists or natural processes.

Forsythe et al. (2008) demonstrated that familiarity influences the extent to which we perceive an object as complex or as simple. This suggests that the prevalent FD value of the environment in which we grow up may also impact upon the extent to which we find images aesthetically pleasing. The anthropologist Lowie (1921) laid the groundwork for exploration of universals in aesthetic responses by examining the decorative artistic and abstract style of parfleches of the Crow and Shoshoni tribes. In comparing the proportions of the geometric shapes to that of the “golden section” proportion (Fechner, 1876), Lowie found neither group demonstrated the measurement as a universally preferred proportion, rather the Shoshoni norm fell above Fechner’s proposals, and Crow ratios fell below. Cross-cultural work continued to grow in the 1950’s with findings demonstrating high correlations in aesthetic responses between Australian Aboriginals and Caucasian participants (McElroy, 1952). However, authors began to raise concerns over the existence of universal aesthetic principles, arguing that beauty could more reasonably be determined by culture. Researchers, including Lawlor (1955), began to credit cultural experience as an overpowering component in understanding aesthetic universals that may exist when looking between two or more very different cultural heritages. Large scale cross-cultural differences have been demonstrated in judgments of geometric illusions. Some included samples from up to 15 societies over a period of 6 years (Segall et al., 1966) and non-western cultures would be differentially susceptible to geometric illusions because they have discovered different visual habits that may produce/inhibit particular illusionary responses. Similar findings were reported between native Arctic and non-native Arctic workers, and those with no Arctic experience (Sonnenfeld, 1967), once again suggesting that landscape preference was a result of culture mediated preference.

Comparisons of art experts in Japan and the United States have demonstrated that whilst there is some cross cultural agreement in *expert* aesthetic evaluation (Iwao and Child, 1966), as a whole evidence points towards universal aesthetic exceeding the bounds of “culture” as we classify it (Child and Siroto, 1965; Ford et al., 1966; Child and Iwao, 1968; Iwao et al., 1969). Soueif and Eysenck (1971) and Eysenck and Soueif (1972) recruited British and Egyptian art students and lay people (non-art trained participants) to explore aesthetic responses. Participants were asked to rate Birkhoff’s (1933) polygons for pleasantness. Results showed interesting differences between cultural responses to these shapes. British art students showed preference for simple figures, and British lay people preferred complex figures. This trend is reversed within the Egyptian sample with art-educated participants preferring the complex figures and the lay participants demonstrated preference towards the simple images. Despite this curious directional result, no significant differences were found between the British and Egyptian groups as a whole. There were also no significant differences in preference between both art and non-art trained participants, although the trend seemed to suggest reversed trends for complexity preference. Eysenck and Soueif (1972) did not believe that their data support considerably large differences in aesthetic preference between both cultures but instead hint towards more universal preferences over cultural issues.

Studies examining sub-cultural or micro environmental differences in Australia, Pakistan and Thailand have reported variability within sub-cultures (Anderson, 1976). The results show general consistency between preferences, but marked differences based on demographic details and background. For example, within the Australian sample, preferences of participants from a suburban environment and school differed significantly from participants from an urban and industrialized environment and school. Similarly, Zube and Pitt (1981) and Zube (1984) found differences between Yugoslavian, West Indian and American cultures that suggested that not all cultures shared a preference for natural scenes over manmade.

## Aging and Gender

Aesthetic responses to natural environments have been found to show marked age differences (Balling and Falk, 1982). Children showed preferences for Savannah scenes over more familiar environments. Adolescences demonstrated consistently lower preferences for all scenes compared to any of the other groups in the sample. This effect has been reported elsewhere with adolescent preference for natural scenes distinctively lower when compared to younger or older generations (Herzog et al., 2000). Adolescents show a higher appreciation of developed and urban settings than their different aged counter parts (Lyons, 1983; Medina, 1983, unpublished doctoral dissertation; Kaplan and Kaplan, 2002). Kaplan and Kaplan (1989) argue that preference does not suggest adolescents do not show appreciation for natural settings, rather the pattern of preference related to the adolescent’s relationships with urban spaces. Such spaces facilitate group and social spaces, rather than solo and natural scenes.

Elderly samples have been found to display relatively low preference for wild nature over more managed and developed natural landscape (Balling and Falk, 1982; Lyons, 1983; Strumse, 1996; Van den Berg and Vlek, 1998). Some have attributed this difference in elderly samples as relating to evolutionary drives that would make wilderness scenes a larger risk to vulnerability, both physical and psychological. Alternative theories such as increased cultural and experiential shaping have been attributed to this change in preference at older ages (Van den Berg and Koole, 2006). However, studies of non-normal aging appear to show stability in preference for art stimulus despite neurodegenerative diseases such as Alzheimer’s, which suggests that in older age preferences remain stable despite the functioning capacity of working memory (Halpern et al., 2008). Other studies exploring neurological conditions however appear to show aesthetic changing in abnormal ageing such as a marked aesthetic difference in art production and quality following a stroke (Zaimov et al., 1969). The impact of chronological age alone is difficult to interpret as a separate influential factor in aesthetic judgments because individual experience, education and social factors cannot be uncoupled from age.

Gender could be mediating factor in preference, but the limited research that exists is split with reports of clear aesthetic differences, or no tangible evidence what so ever (Farrell and Rogers, 1982; Limbert and Polzella, 1998). Earlier studies demonstrated that women appeared more attracted to impressionist paintings than men, with men showing preference for modern paintings (Bernard, 1972). Women preferred representational art, which displays soft and curved patterns whereas men preferred more abstract work containing higher numbers of pointed or sharp shapes (Cupchik and Gebotys, 1988). However, given that these studies were reported almost 30 years ago, it is possible that such differences have diminished. More recent studies have reported that women demonstrate an overall higher appreciation of art than males (Frumkin, 1963) and that abstract art was generally rated more highly by females than their male counterparts (Furnham and Walker, 2001). Polzella (2000) looked at gender differences in college students for color reproductions of art stimulus. Results found that impressionist paintings were judged as the most pleasing by females, and also evoked relaxation and alertness. It was concluded that the differences between males and females might be a result of differences in perceptual style and emotional sensitivity between genders (Polzella, 2000). It appears that perceptual styles or affective processing may result in significant differences in aesthetic response between genders. For example, Fedrizzi (2012) found that paintings that captured behavior evoked more pleasure and attention among female participants over male participants. Fedrizzi (2012) suggests that neuroanatomical studies can enhance the comprehension of why such gender differences appear to exist. Other evidence demonstrating gender differences in cognitive processes (Leder et al., 2004; Marin and Leder, 2013) supports this claim. Cela-Conde et al. (2009) also report gender-related differences in parietal activity during aesthetic appreciation and judgments. While in both sexes activity is focused in the parietal lobe, it appears that males show lateralized right hemisphere activation in the parietal lobe while females show bilateral activity in the same region. These results point towards a difference in the way males and females process aesthetic appreciation, although specifying how the differences manifest in response is challenging and yet to be explored in great depth.

## The Current Study

Given the visual similarity and theoretical links between FD and visual complexity, this article aims to first measure the relationship between the FD of the stimulus set and visual complexity. Overall patterns of preference were identified and following on from this, linear mixed-effect modeling was used to investigate the extent to which individual differences of culture, gender and age account for variation in preferences. The analysis explored two different models to map and understand patterns of preference. The first model tested a linear complexity model (likelihood of selecting the more complex image from the pair of images) and the second a mid-range FD model (likelihood of selecting an image within the mid-range 1.3–1.5D from the pair of images).

### Stimulus Measurement

**Figure 4:**
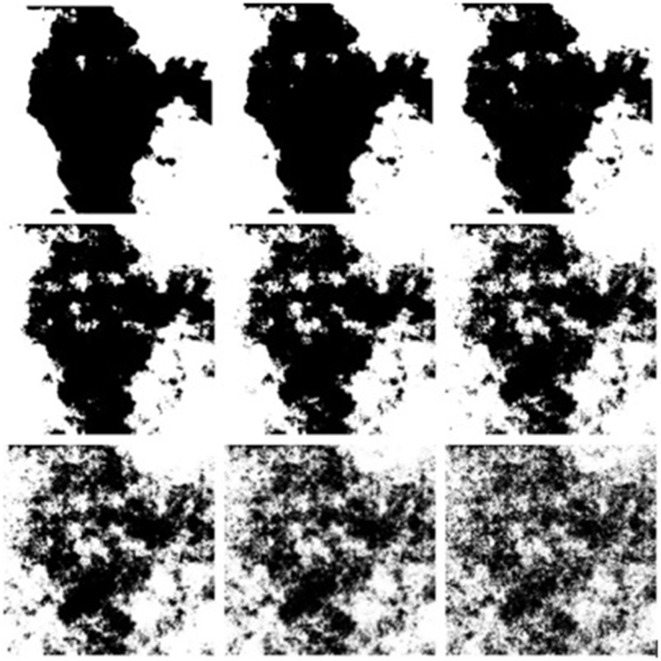
**Example sample set of a full fractal range (top left through to bottom right D1.1–D1.9 increasing in 0.1 increments)**.

The stimuli consisted of 81 abstract monochrome fractal images (9 full sets of 9 iterations of FD) generated using the mid-point displacement technique (Fournier et al., submitted). All stimuli were developed in bitmap format (Figure 4).

Initial stages of the article involved obtaining visual complexity scores for 81 abstract fractal patterns by analyzing for visual complexity using GIF compression based on measures by Forsythe et al. (2003). Forsythe et al. (2008, 2011) used compression file size as a measure for visual complexity. This measure works well on highly complex images such as photographs and drawings. Given the nature of the monochrome images in this study, bitmap compression to converted Gif file ratio was applied. The amount of information between the original bitmap file and new compressed file provides the GIF Ratio score. Higher GIF ratios represent lower complexity scores. Higher GIF ratio scores are a result of less difference between the bitmap size and the GIF compression size.

The results of this exploration show a strong negative correlation between FD and the GIF ratio complexity measure (*r*_(79)_ = −0.93, *p* < 0.01) were found for the 81 image set. In other words, images with small compression ratio scores tend to have a higher FD. This allows assumptions about preferences to both FD and visual complexity to be made in subsequent analyses.

### Experiment

#### Procedure

Participants were asked to provide aesthetic responses to the stimulus set, standardized for complexity. Using a two alternative forced choice analysis, participants made preference choices between 57 stimulus pairs. To run a full forced choice analysis it would have involved running 6480 pair comparisons. The 57 pair selection was based on work by McManus (2009) who developed a regression-based technique for reducing the numbers of pairings required when using a forced choice design in visual aesthetics. In brief, McManus’s method samples an entire range of stimuli but also provides detailed information on closely similar images. This design is further justified by Taylor et al. (2011) who found no significant differences within closely related sets of fractal images.

In addition to preference choices, participants also provided demographic details including Age, Gender, and continent of residence to allow exploration of individual differences in preference responses.

Ethical approval for the study was granted by the Ethics Committee in the Institute of Psychology, Health and Society at the University of Liverpool.

#### Participants

Four hundred and forty-three participants, 228 men and 204 women, aged between 17 and 88 (*m* = 31.03 *SD* = 14.45) took part in the study. Participants were recruited using a variety of methods including opportunity, online and targeted recruitment. Participants were recruited across four continent locations, Europe (*n* = 177), North America (*n* = 24), Central Asia (*n* = 195) and Africa (*n* = 97). Each participant was provided with an information sheet ahead of the study and informed consent was recorded for each.

#### Results

To explore the results in depth three stages of analysis were conducted. Initially, overall patterns of preference were explored using SPSS software package (1a) followed by the Complexity Model Linear Mixed Effects analysis (1b) and finally the Mid-Range Model, Linear Mixed Effects Modeling (1c) using the software environment R.

### Experiment 1a: Exploring Preference Patterns

**Figure 5:**
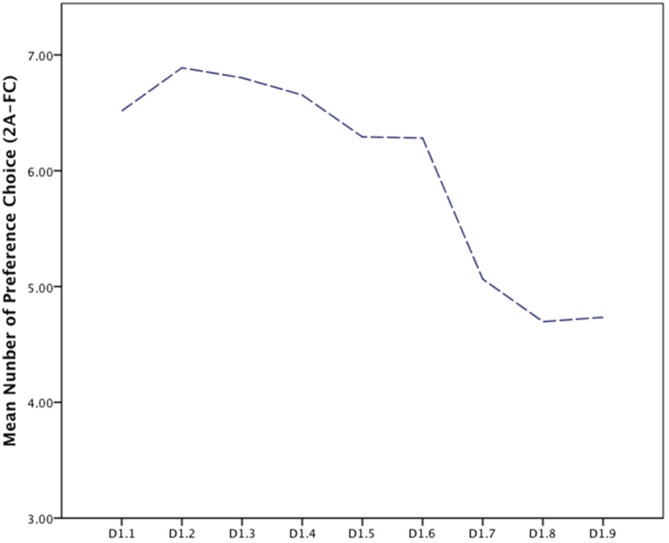
**Line graph of preference choice number (2A-FC) for fractal dimension (FD) for overall sample**.

Preference patterns across the scale of the averaged FD were explored for frequency of choice (Figure 5). Overall patterns of preferences found the peak of preference at D1.2 (*M* = 6.89, *SD* = 3.89) and preference choices lowest at D1.8 (*M* = 4.69, *SD* = 3.89). Mauchly’s test indicated that the assumption of sphericity had been violated, ($\chi_{\left(35\right)}^{2}$ = 5518.76, *p* < 0.001). Therefore degrees of freedom were corrected using Greenhouse-Geisser estimates of sphericity (ε = 0.18). The results show that there was a significant effect of FD, (*F*_(1.467, 777.46)_ = 35.06, *p* < 0.001, $\eta_{p}^{2}$ = 0.06). These results suggest that preference ratings differ significantly between each FD supporting an exploration of preference patterns in a number of directions (Figure 5).

#### Location

**Figure 6:**
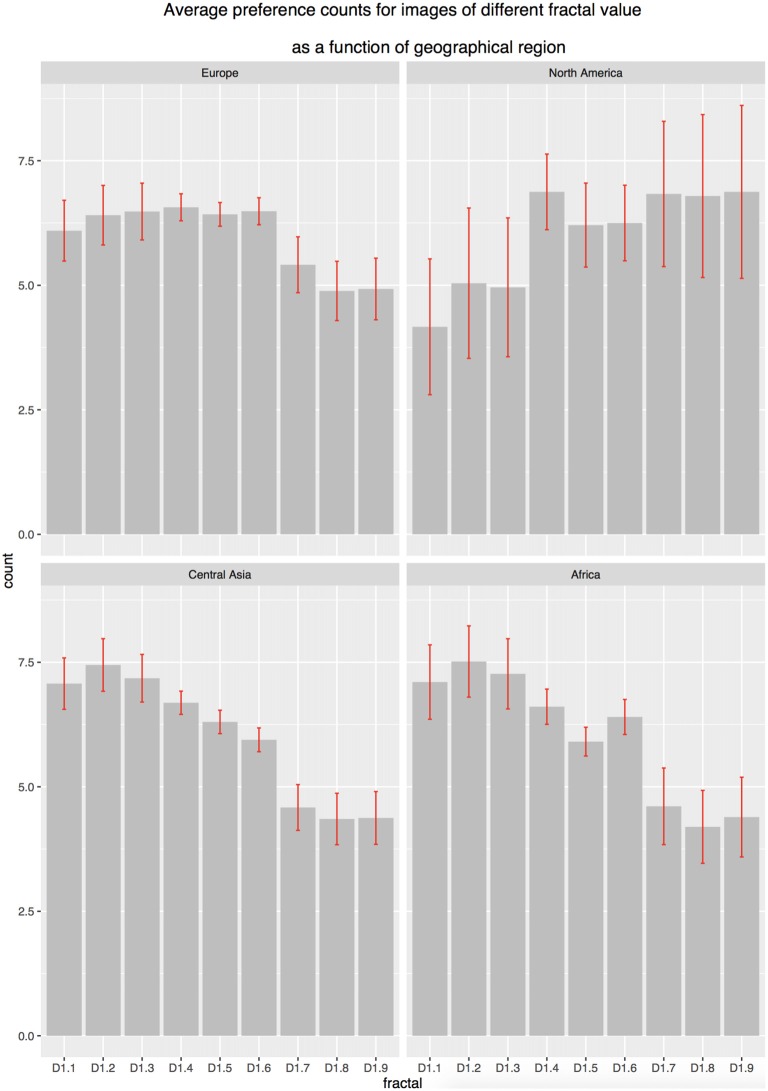
**Bar chart of preference choice number (2A-FC) for FD as a function of geographic location (error bars 95% CI)**.

To explore the role of individual differences in preference for FD analysis we examined mean preference choices across the fractal scale between continent location groupings. Mauchly’s test indicated that the assumption of sphericity had been violated, ($\chi_{\left(35\right)}^{2}$ = 4995.552, *p* < 0.001). Therefore, degrees of freedom were corrected using Greenhouse-Geisser estimates of sphericity (ε = 0.184). The results show that there was a significant effect of FD and location grouping (*F*_(4.42,720.6)_ = 4.00, *p* < 0.001, $\eta_{p}^{2}$ = 0.02). These results suggest that preference ratings differ significantly between FD and location grouping. However, *post hoc* analysis found no direct differences across the groups (Figure 6). Within Figure 6, error bars demonstrate significant differences between the samples with the North American samples demonstrating significantly greater variance in findings. The North America sample also shows a negative linear preference differing from the suggested positive direction of preference noted in the European, Central Asian and African samples. This finding may be a result of the limited participant numbers in this group.

#### Gender

**Figure 7:**
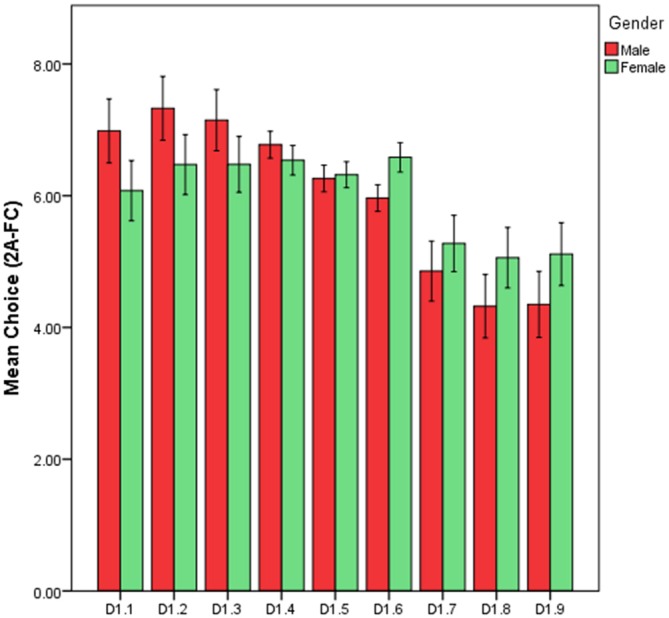
**Bar chart of preference choice number (2A-FC) for FD as a function of gender (error bars 95% CI)**.

The frequency preference patterns of FD were explored across gender. Male preference peaks at D1.2 (*M* = 7.32, *SD* = 3.94) and continues to fall as FD increases from this point. The patterns of preference for male participants point towards a low-to-mid peak in preference for fractal complexity. The female sample shows a different pattern of preference across the fractal scale. Female preference peaks at D1.6 (*M* = 6.58, *SD* = 1.88) and analysis shows less variation across each FD than male participants. This lack of clear variance across scales means there is no clear directional or curvilinear pattern emerging from the frequency data for the female sample (Figure 7). Additional *post hoc* analysis explored if gender preference differed as each FD as a function of gender. Results show no significant difference in preference across D1.4 (*t*_(528)_ = 1.52, *p* = 0.129), D1.5 (*t*_(528)_ = −0.404, *p* = 0.687) and D1.7 (*t*_(528)_ = −01.32, *p* = 0.187) however significant differences were found across D1.1 (*t*_(528)_ = 2.68, *p* < 0.01), D1.2 (*t*_(528)_ = 2.53, *p* < 0.05), D1.3 (*t*_(528)_ = 2.11, *p* < 0.05), D1.6 (*t*_(528)_ = −4.02 *p* < 0.001), D1.8 (*t*_(528)_ = −2.18, *p* < 0.05), and D1.9 (*t*_(528)_ = −2.17, *p* = < 0.05).

### Experiment 1b: Complexity Model (A) Results

Two models of preference were tested using linear mixed effects analysis. The first of two models explored was a Complexity Model, which explored the extent to which the variables, Continent, Gender and Age could predict the choice of more complex (higher FD) fractal images, from a pair. The complexity model accounts for significantly more variance with fixed and random effects (AIC = 17040, *df* = 21) than the null model with random effects alone (AIC = 17154, *df* = 3), suggesting that the model is improved with the additional variables ($\chi_{\left(18\right)}^{2}$ = 125.14, *p* < 0.001). Additional goodness of fit analysis found that Continent ($\chi_{\left(15\right)}^{2}$ = 99.06, *p* < 0.001) and Gender ($\chi_{\left(7\right)}^{2}$ = 29.29, *p* < 0.001) significantly improves the overall fit of the model. However, age ($\chi_{\left(7\right)}^{2}$ = 6.845, *p* = 0.445) does not significantly add to the overall prediction of the model.

**Figure 8:**
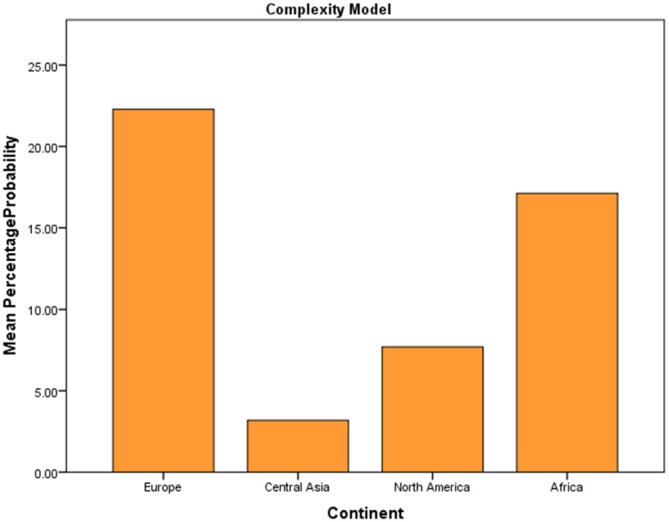
**Bar chart of percentage choice of complex image from a pair across continent**.

Results of the model identify a significant main effect of continent on preference for complex fractal patterns. European participants show an average 22% choice for the complex image from a pair (Figure 8). This did not differ significantly from the African sample (*β* = −0.356, *z* = −0.535, *p* = 0.592), with an average choice of approximately 17%. Significant differences were however found between European (22%) and North American (8%) samples (*β* = −1.311, *z* = −2.601, *p* < 0.01) and European (22%) and Central Asian (3%; *β* = −2.261, *z* = −8.267, *p* < 0.001) suggesting individual differences in preference across locations. Additional main effects were also found for gender, with females showing a significant increased choice in complex fractal shapes (20%) compared with males (5%; *β* = 1.585, *z* = −4.827, *p* < 0.001).

**Figure 9:**
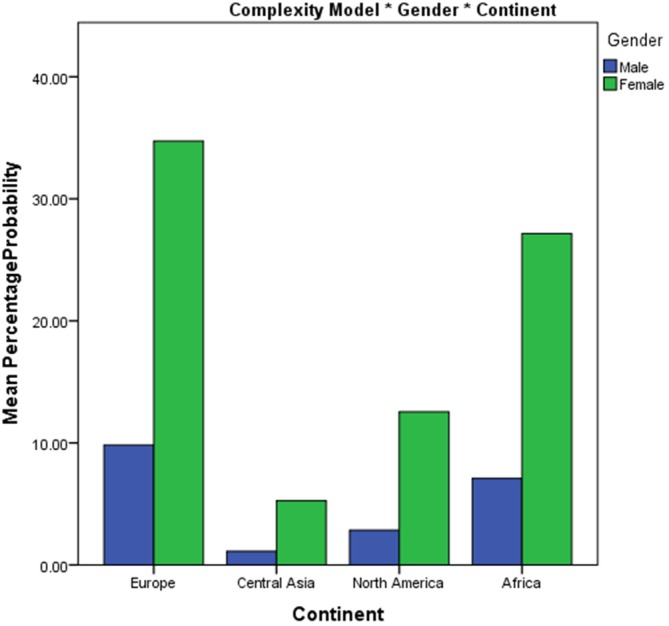
**Bar chart of percentage choice of complex image from a pair across continent and gender**.

**Figure 10:**
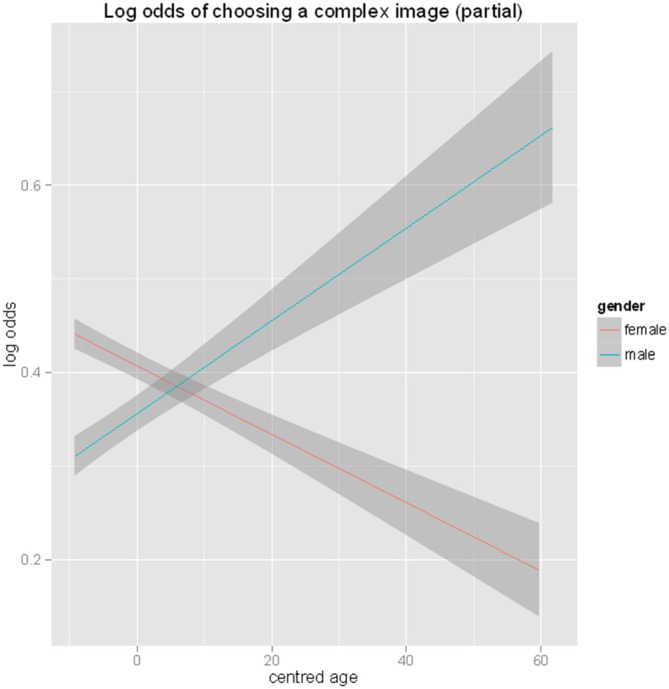
**Interaction of percentage choice of complex image between gender and age in complexity model**.

Although the difference between the African and European continents was not significant as a main effect, the model shows a significant interaction between continent (Africa-Europe) and gender (*β* = 1.446, *z* = 2.64, *p* < 0.01). African males show a lower probability of choosing a complex image (7%) than African females (27%), and European males show a lower probability (10%) than European females (35%) of choosing the more complex image from a pair (Figure 9). Interaction effects were also found between Central Asia and Europe and Gender (*β =* 1.446, *z* = 2.636, *p* < 0.01). As discussed, European males show approximately 10% probability with European females, having approximately a 35% probability of choosing the more complex image from a pair. Central Asian males had an approximately 1% probability of choosing the more complex image and females had a 5% probability of choosing the more complex image. A significant interaction was also seen between gender and age (*β =* 0.028, *z* = 2.096, *p* < 0.01). Male and female complexity choices show changes as a function of age. Female participants’ preference for complexity decreases with age, whereas male participants’ preference for complexity increases with age (Figure 10).

### Experiment 1c: Mid-Range Model (B) Results

The Mid-Range Model explored the extent to which the variables Continent, Gender and Age could predict the choice of the Mid-Range image from a pair. The Mid-Range Model accounts for significantly more variance with fixed and random effects (AIC = 17040, *df* = 21) than the null model with random effects alone (AIC = 17154, *df* = 3), suggesting that the model is improved with the additional variables ($\chi_{\left(18\right)}^{2}$ = 125.14, *p* < 0.001). Additional goodness of fit analysis found that Continent ($\chi_{\left(15\right)}^{2}$ = 27.89, *p* < 0.05) improved the overall fit of the model. However, because the chi-square test based on likelihood ratios is anti-conservative and the rule-of-thumb is to halve the *p*-values (Pinheiro and Bates, 2000), this improvement can only be considered marginally significant; the variable Age marginally improves the model, but not significantly ($\chi_{\left(7\right)}^{2}$ = 12.99, *p* = 0.072) however Gender ($\chi_{\left(7\right)}^{2}$ = 11.81, *p* = 0.107) does not significantly add to the overall prediction of the model.

**Figure 11:**
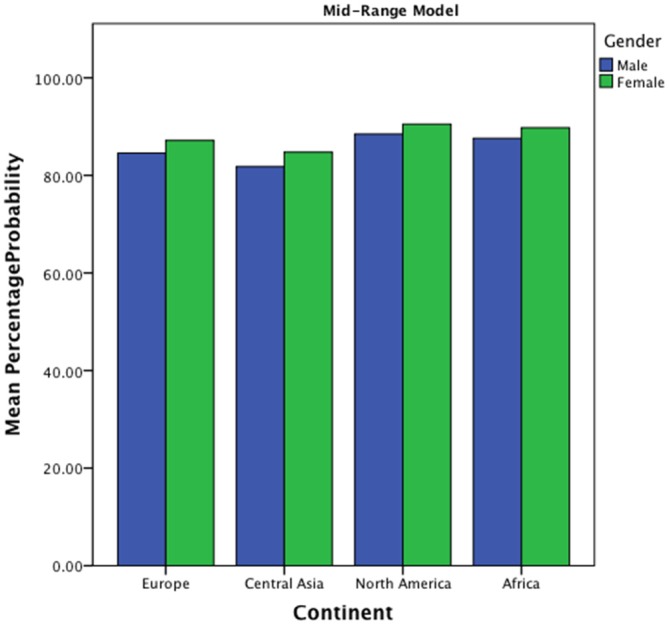
**Bar chart of percentage choice of mid-range image from a pair across continent and gender**.

The model found a main effect of continent on preference for mid-range fractal image. Significant differences in percentage choices were seen between Europe and Central Asia (*β =* −0.198, *z* = −2.262, *p* < 0.05). European samples have an average probability of choosing the mid-range D of 85% and Central Asia samples show an average 83% probability. There are also marginally significant differences in preference choice between European and North American samples (*β* = −0.339, *z* = 1.937, *p* = 0.052). European samples demonstrate a lower probability of choosing mid-range fractal patterns (85%) than North American samples (89%). In addition, the model found a significant main effect of gender on preference for mid-range fractal images with Females (88%) showing an increased probability of choosing mid-range fractal patterns when compared with males (86%; *β* = −0.215, *z* = −2.025, *p* < 0.05). Significant interaction was seen between continent (Central Asia-Europe) and gender (*β* = 0.299, *z* = 2.242, *p* < 0.05), with European males showing a lower preference choice (85%) than European females (87%), and Central Asian males showing a lower choice (82%) than females (85%; see also Figure 11).

## Discussion

Models of complexity and preference can be traced back to the very early field of empirical aesthetics (Birkhoff, 1933). It has been repeatedly noted that complexity plays a role in aesthetic judgments. There have however been inconsistent findings within the field, which may be explained by the associated difficulty in defining visual complexity as one construct. As such the current article aimed to unpick the multifaceted construct of visual complexity and explore the statistical relationship between computational complexity and FD. As noted by Taylor et al. (2011), most complexity research has focused on the responses to complexity of scenes made up of Euclidean shapes, suggesting that the applicability of previous findings to natural scenes are limited. Here we attempted to further develop this field by first examining the construct of complexity and FD and then exploring the individual factors that can predict preference towards these patterns**.** Our results demonstrated the hypothesized relationship between constructs and also found an influence of individual differences across environment (continent) and gender in complex fractal preference.

In models of both visual complexity and fractal aesthetics it appears mid-range fractal images have a powerful aesthetic draw. Our aim was to explore the stability of the mid-range preference and examine the claims that preferences exist universally for this mid-range (Berlyne, 1970; Spehar et al., 2003). This was achieved by examining patterns of preference across Culture, Gender and Age. When examining participant responses as a whole, data reported here corroborate previous studies (Taylor et al., 2011) that demonstrate a preference for mid-range FD. However, constituent analysis highlights the potential issues with the current theory of mid-range fractal peak in preference, as culture, and gender of participants appears to play a role in shaping different patterns in preference.

### Testing the Mid-Range Hypothesis

The Mid-Range Model found evidence to suggest individual differences account for the variance in preference for mid-range fractal images. Main effects of location were shown with significant differences in preference between European and North American samples. North American participants showed a heightened preference for mid-range images. Females demonstrate a heightened preference choice of the mid-range fractal images. Interaction effects are evident across gender and location (Central Asia—Europe) with females more often choosing the mid range images, than their male counterparts.

Initial frequency choice analysis shows the overall patterns of preference demonstrate a peak at D1.2, one point below the previously reported (D1.3–1.5) FD peak (Taylor et al., 2011). The pattern emerging is one of heightened preference for the low to mid-range fractal patterns. This pattern is somewhat consistent with previous findings, however here we find that preference falls incrementally with increases in FD. This suggests that as FD increases preferences decrease.

Looking at the overall preference patterns in the data, there is an evident negative relationship, with groups demonstrating higher preference choices towards the lower end of the fractal scale and preference decreasing as FD/complexity increases. Overall preference patterns show a complex story. When the entire population is examined, samples predictably show distributions consistent with the mid-range fractal hypothesis, however the variability of preference, as a function of location and gender, does not support the theory of universal preference for mid-range fractal patterns.

Results from the Linear Mixed effects mid-range model confirm this pattern with high overall preference choice percentages found in the choice of mid-range over non-mid range images (80–90%). One potential rationale could be the survival benefits associated with landscape displaying approximately mid-range FD. Orians (1980) Savannah hypothesis suggested that spontaneous emotional responses to landscapes that are positive for survival or instincts and then preference fall for Savannah type landscape (found to display mid-range fractal patterns) because they are most akin with the environment in which we developed and provided shelter and survival benefits. The Savannah hypothesis has been found to be consistent and preferences for Savannah-type landscape are seen in individuals, even if they have never had visual interactions with this type of environment (Falk and Balling, 2009). This effect is particularly strong in children (Balling and Falk, 1982). Similarly, the refuge theory rationalizes that preference for visual environment is greatest for those that can offer both shelter and ability to move undetected (Appleton, 1975). Although the refuge theory has been criticized for its limited ability to demonstrate why differences exist across culture, Appleton (1996) addressed these criticisms when he asserted that preferences may well be shaped by culture, experience and historical influence. He highlights that the foundations of these trends are not within a vacuum and as such still have links in environmental preferences based upon these evolutionary instincts of aesthetic preference. While it could be suggested that biological and evolutionary foundations play a role in shaping our visual preferences for fractal patterns, they could also be shaped in part by our current visual and cultural experiences as found in some previous cross-cultural research, accounting for significant interactions found here.

Aesthetic responses to symmetry can also show how preference for abstract geometric forms can be based on evolutionary survival. Symmetry appears in mate selection as it represents strong genes, or may have been an unintentional by-product of visual shape recognition (Enquist and Arak, 1994). Symmetry is an easily perceived measure of genetic quality (Møller, 1990; Parsons, 1992) and these same conclusions can be drawn about responses to fractal patterns. FD at the mid-range is an effective and easily perceived measure of naturalness and has the most positive psychological and physiological benefit (Taylor et al., 2011). Similarly, preferences for facial attractiveness are found in early infants before learned behavior could be possible, suggesting innate and evolved responses (Langlois et al., 1987). As our experiences with faces grow we begin to understand society specific visual experiences what the “average” face looks like and respectively our preferences for faces become more socially and culturally tied. Using this theory as a guide, we could propose a type of aesthetic development for fractal patterns. Innate responses could (and should) be found toward mid-range, but with repeated exposure to other non-mid or non-fractal environments what we consider most aesthetically pleasing will change with experience. Cultural ties reduce and change a typical scene to which we compare new viewing. We eventually learn the “norm” of the visual environment in which we spend time and these repeated exposure shaped our aesthetic perceptions according to the exposure and fluency hypotheses (Zajonc, 1968; Reber et al., 2004).

### Gender

Despite the consistent patterns emerging that support universal patterns of preference for images falling within the mid-range of the fractal spectrum when exploring between subjects, gender played on a role in the likelihood and patterns of preferences shown toward this range. Males demonstrate a negative linear relationship between preference choice and FD with an incremental decrease in preference as FD increases. Females, however, show a higher peak preference point (*D* = 1.6) with less variance across the FD scales than the male sample. Linear Mixed Effects modeling suggests that gender is a significant predictor of preference for mid-range fractal patterns. Females have a significantly higher probability of choosing mid-range patterns over males.

### Testing the Complexity Hypothesis

Findings discussed above outline the patterns found when exploring the universality of the mid-range hypothesis. However, the study also attempted to explore the links between FD and visual complexity and in addition attempted to shed some light of the patterns of preference for complex stimulus, which is currently unconfirmed. While Berlyne (1970) proposed a curvilinear pattern, Forsythe et al. (2011) proposed a linear relationship between preference and complexity. This stalemate suggests further factors may influence how these preferences emerge.

Compared with the likelihood of selection for the mid-range images (approximately 80–90% of choices for the mid-range), there were significantly lower levels of selection of images of higher complexity with selection rates for the complex image from a pair ranging from less than 1% up to 20% selection. Samples demonstrated less preference towards complex images. Compared with the mid-range model, the complexity model found significant individual differences, which appear to shape preference for complexity stimulus. These include location (continent) and gender and point toward a significant role of individual differences when exploring aesthetic response to complexity.

### Location

When exploring across location there are emergent differences in preference patterns. The European, Central Asian and African samples show similar patterns of preference with peaks at the lower-to-mid end of the fractal scale and preference dropping incrementally as FD (and related complexity) increase. The lowest preference choices in this sample are seen in the high end of the fractal scale. The North American sample demonstrates a different pattern of preference, with highest preferences shown for higher FD. Within this sample (the smallest populated sample) preference peaks at D1.4 and is lowest across the lower FDs (*D* = 1.1–1.3).

Analysis of a worldwide sample demonstrated significant differences in the frequency patterns of preference as a result of locational grouping, suggesting that residential location shapes preferences for fractal patterns. These differences were also found in data interrogating both the complexity and mid-range models, suggesting that the location in which we spend time influences the preference choices that we make. North American participants have the highest choice of mid-range fractal patterns. European samples are three times more likely than the other continents to choose complex fractal patterns.

### Gender and Continent Interactions

There are significant interactions between continent and gender in the complexity model, with females more than three times more likely to choose the complex image than their male counterparts. This pattern reverses with aging; with male preference for complexity increasing and female preference decreasing. This raises interesting questions about the stability of preference across age and other individual differences.

In addition to the main effect of continent on fractal complexity preference, continent was also found to interact significantly with gender. Whilst European participants are generally more likely to choose the complex image, there are different directional patterns in probability with males in Central Asia being more likely to pick the complex image and European females being more likely to pick the complex image. The pattern was somewhat different for African/European participants, with female participants being most likely to choose the complex image than males in both continent groups. These results demonstrate that it is possible to predict preference based on continent and gender although the relationship is a complex one. These cross-cultural findings contest many of the previous studies that report a general consistency in aesthetic response across cultures (Child and Iwao, 1968; Iwao et al., 1969; Eysenck and Iwawaki, 1971) and suggest that visual experiences over universal preferences may play a larger role when making aesthetic judgments on complex images.

## Conclusion

Berlyne (1970) proposed a curvilinear hypothesis of aesthetics and complexity, in which preference increases linearly with complexity until an optimum level of visual arousal is reached, at which point preference begins to fall. Despite its wide spread recognition, Berlyne’s arousal theory has been criticized as it fails to predict the point of preference (Krupinski and Locher, 1988; Martindale et al., 1990). Questions have also been raised about issues such as the samples size and the existence of a familiarity bias (Forsythe et al., 2011). Alternative linear patterns of preference have been proposed (Forsythe et al., 2008, 2011) suggesting that further work is required to understand the relationship between beauty and complexity. The Complexity model within this article explored the linear model of complexity, examining if individual differences (Culture, Gender and Age) shaped the likelihood of choosing a complex image from a pair. Results demonstrate significant main effects of gender, with females demonstrating higher preference for complex images than males and a main effect of continent across European and Central Asian, and European and North American samples. European samples show overall a higher preference for fractal complexity. An interaction between gender and age was also discovered within the model and results show an opposite directional effect with male participants’ preference for complexity increasing with age and female participants’ preference for complexity decreasing with age.

Speculations could be made based on the results from the study to offer support for the hunter-gather hypothesis that differences in spatial abilities between genders provide a convincing scenario of sex differences based in our evolutionary history, as a result of the division of labor between sexes (Silverman and Eals, 1992; Silverman et al., 2007). Although no neuropsychological measures were used in the current study, differences in neural activity in gender have been noted (Cela-Conde et al., 2009). Viewing scenes has been proposed to differ as a result of the labor division between hunting (a primarily male activity) requiring coordinated spatial relation processing and mental rotation skills and foraging (a mainly female pursuit) requires categorical spatial relations, including recognition and remembering the content of varied objects and spatial relations between the objects (Silverman and Eals, 1992; Silverman et al., 2007). Could it then be argued that the differences in neural processes are a result of the different visual strategies? Foraging has been proposed to require a greater understanding of the complex visual scenes, therefore this could account for the heightened preference in females for high complexity images over simpler patterns as it indicates signs of vegetation. Females employ categorical spatial relations during aesthetic judgment and this processing relies heavily on long-term memory so associations can be made between previous and current scenes. Suggestions could tentatively be made that if long-term memory plays a greater role in women than men, aesthetic judgment particular for scenes, woman use memory more that could mean that experiences play a larger role in aesthetic processing than it does in males.

The current study adopted a dichotomous forced-choice design with pure fractal imagery. Whilst such design permits control of familiarity effects, color and other collative variables it is not ideal for understanding the nature of real world preference. It is further acknowledged that macro visual environment across continent may have some, but limited, differences across the locations used. As direct visual environment appears to play a role in shaping preference patterns, a fruitful area to explore would be the effect of micro or sub-cultural environments, for example urban and rural landscapes. Such analysis would allow a further investigation of the role that exposure to fractal over Euclidean geometry has in shaping preference.

## Author Contributions

NS: Work from her PhD. AMF: Supervisor and co-writer. RR: Statistical guidance. RT: Fractal advice. MH: Data collection in Egypt.

## Conflict of Interest Statement

The authors declare that the research was conducted in the absence of any commercial or financial relationships that could be construed as a potential conflict of interest.
